# Retraction: Melioidosis: Clinical impact and public health threat in the tropics

**DOI:** 10.1371/journal.pntd.0008441

**Published:** 2020-07-01

**Authors:** The PLOS Neglected Tropical Diseases Editors

Following publication of this article [1], concerns were raised to the journal that some information reported in the article is inaccurate and/or incomplete, and that recommendations discussed in the article are not supported by current guidelines for melioidosis. In addition, concerns about the review article were discussed in a Formal Comment published in 2019 [2].

The authors confirmed there were numerous errors in the article. The *PLOS Neglected Tropical Diseases* Editors-in-Chief and members of the *PLOS Neglected Tropical Diseases* Editorial Board evaluated the article and the issues raised, and determined that this review includes unreliable information; that statements regarding clinical diagnosis and treatment are not adequately supported by published findings; and that in light of these issues, the review could introduce potential risk to patient care.

Specific issues confirmed in the editorial assessment include the following, which are discussed in more detail in [2]:

- In the section, ‘Antibiotic resistance and susceptibility/treatment of melioidosis,’ the characterization of the resistance and susceptibility of *Burkholderia pseudomallei* to antibiotics is inaccurate.
- The section, ‘Laboratory diagnosis’ includes inaccurate and incomplete information that does not reflect current guidelines and knowledge in this area. Critical information regarding the interpretation of diagnostic results is missing.
- The discussion of treatments includes several statements that are not supported by randomized controlled trials, or in some cases, that do not align with clinical practice standards in place at the time of the publication of the review. Statements of concern include those discussing the standard oral treatment, the utility of third-generation antibiotics, and the efficacy of combination treatment (chloramphenicol, doxycycline, and trimethoprim-sulfamethoxazole), fluoroquinolones, and immunotherapy.
- The discussion of geographical distribution, disease incidence, and ecology, does not discuss some key articles that were published prior to the review.
- Table 1 (“Global distribution of melioidosis outbreak, incidence, and their reported cases (an overview)”) does not include all countries where melioidosis is present and does not accurately report the number of published cases per country in some cases.
- The section, ‘Development of antibodies and vaccines for prevention of melioidosis,’ lacks information about relevant advances.

The *PLOS Neglected Tropical Diseases* Editors-in-Chief have determined that this review article is unreliable in light of the above issues, and we are therefore retracting this article. We regret that these issues were not addressed during the article’s pre-publication peer review.

RPS, GS, and LHKL disagreed with retraction. BGS either did not reply directly or could not be reached.

LHKL apologized for the errors in the article. RPS commented that the issues in this case could have been addressed through revisions had they been raised prior to the article’s publication.
